# How Women with Endometriosis Use Social Media for Support and Self-Management: An Analysis of Reddit Content

**DOI:** 10.3390/ijerph22111706

**Published:** 2025-11-11

**Authors:** Alaina Loughran, Kirstie Daken, Carol du Plessis, Amy B. Mullens

**Affiliations:** School of Psychology and Wellbeing, Centre for Health Research, University of Southern Queensland, 11 Salisbury Road, Ipswich, QLD 4305, Australia

**Keywords:** endometriosis, Information–Motivation–Behavioural (IMB) skills model, Reddit, self-management, women

## Abstract

Women with endometriosis often experience insufficient knowledge and support regarding their condition within the healthcare system. Women use social media to obtain information, share personal experiences, and establish connections with others. This study examined how women with endometriosis-like symptoms use Reddit to seek support and acquire knowledge for self-management, with three research questions guided by the Information–Motivation–Behavioural skills model. A total of 194 threads were analysed from the r/endo subreddit, and a reflexive thematic analysis was conducted. Three overarching themes were identified (information, motivation, and behavioural skills) and six subthemes emerged from the data (endometriosis experience, endometriosis medical care, personal life impacts, social existence impacts, management strategies negative, and management strategies positive). Findings highlight that Reddit functions as a source of shared knowledge, symptom validation, emotional support, supplementing gaps in traditional healthcare services. This study provides insights into how healthcare systems and professionals can support women with endometriosis, including timely diagnosis and management strategies, in addition to a framework for an endometriosis Information–Motivation–Behavioural skills model. Future research could expand on study findings by implementing this conceptualised model in a mixed-methods context to gain insight into women’s endometriosis experiences within healthcare systems to understand how they can be empowered and supported.

## 1. Introduction

As the World Health Organisation reported in 2025, approximately 190 million women and individuals assigned female at birth worldwide are impacted by endometriosis, including diagnosed and undiagnosed cases [1]. Endometriosis has ramifications for women’s physical and mental wellbeing [2,3], relationships [4,5], education [4], and careers [5,6]. Despite these serious impacts, the condition is currently under-researched [7,8]. Women with endometriosis express feeling they receive inadequate healthcare; specifically, they describe a lack of accessible endometriosis knowledge, and report being “gaslit” about their symptoms by health professionals, leading to psychological distress and feelings of embarrassment [9,10]. Women additionally report finding treatment to be ineffective [10,11,12]. Women thus often feel disempowered and helpless, turning to social media to detail negative interactions within the healthcare system [12,13], obtain support [9,11], share personal experiences [14,15,16], and enhance endometriosis knowledge [12,14,17].

### 1.1. Endometriosis

Endometriosis is a chronic health condition where endometrial tissue grows outside of the uterus, disseminating into neighbouring organs such as the ovaries, bladder, and bowels [18,19,20,21]. Endometriosis causes significant irritation within affected uterine tissue, creating moderate to severe pain during menstruation and often daily, even without menstruation [12,22,23]. Women have described this pain as “excruciating” [24] (p. 1369), [25] (p. 88) and “bodily violence” [10] (p. 5).

The loss of bodily autonomy associated with endometriosis can also impact psychological wellbeing, triggering distress [2,3,26], helplessness [4], depression [27,28], anxiety [27,28], loneliness [10,29], and reduced self-worth [4,30]. Endometriosis additionally impacts women’s social existence [5,24], intimate relationships [31,32], careers and/or education [3,4,5], and financial status [4,5,33]. Furthermore, endometriosis has been linked to infertility in women [9,34]. Of the available endometriosis treatments, the leading form of diagnosis and treatment (laparoscopy) only provides short-term relief from pain [7,35,36]. A laparoscopic procedure may be performed solely to diagnose endometriosis or to both diagnose and treat it [35]. Diagnostic laparoscopy involves visual assessment of the pelvis, sometimes with biopsy, whereas operative laparoscopy additionally includes the removal (excision or ablation) of endometriosis tissue [7,35,36]. Operative procedures are generally more invasive and require a longer recovery period [7,35,36]. Laparoscopies are often inaccessible because of their high cost, particularly for women from financially disadvantaged backgrounds [5]. Despite management strategies such as laparoscopy and pain medication being available, there is currently no cure, or understanding of the cause for endometriosis [37].

### 1.2. The Endometriosis Experience

Women report receiving inadequate care and encountering a lack of knowledge about the condition within the healthcare system [10,12,38]. This lack of knowledge leads women to access alternate sources such as social media to attain appropriate care [11,12]. Research supports women’s experience of minimisation and normalisation of pain within healthcare systems [31,39]. Women express being dismissed and labelled as “dramatic”, and/or having low pain tolerance [4,40,41]. Particularly, research conducted by Young et al. (2019) [42] detailed that healthcare professionals often perceived some women with endometriosis as “difficult patients” (p. 360) and “hysterical” (p. 349). Women frequently experience a long delay in diagnosis, with some suffering from prolonged, often unbearable symptoms for up to 20 years, due to the lack of education and support currently available [10,12]. This invalidation and lack of resources within the healthcare system often causes women to experience heightened psychological distress and feel overwhelmed by a sense of hopelessness [10,11,24].

Due to the current lack of endometriosis understanding, women are frequently misdiagnosed with alternative health conditions including irritable bowel syndrome (IBS), appendicitis, ovarian cancer, anxiety, or depression before consideration of endometriosis, which impacts patient wellbeing, symptom management, and leads to increasing economic burden [4,24,39,43]. Moreover, women report that healthcare practitioners often recommend unsuitable management strategies such as pregnancy or hysterectomy as treatments for endometriosis, with this practice undermining women’s autonomy and agency to have/not have children on their own terms [26,37,39].

### 1.3. Social Media

Existing research suggests that women are accessing endometriosis social media groups on Facebook, Reddit, TikTok, and Instagram for endometriosis-related content due to the perceived lack of support and shortage of accessible endometriosis information within the healthcare system, and because these platforms provide space to challenge sexism and misconceptions related to the condition [40,41,44,45,46,47]. Endometriosis-focused social media pages are perceived as valuable resources, potentially offering information and validation that is absent within the healthcare system [11,13,48]. Social media has become an increasingly prominent platform for engaging with health-related information, support, and advocacy across a range of physical and mental health conditions [33,49,50]. The available empirical evidence indicates three primary reasons why women with endometriosis-like symptoms access social media: for a sense of support and connection, and as a way to share experiences and health information.

Women with endometriosis-like symptoms access social media to receive support from and connect with others who share similar experiences, due to a lack of support and understanding from the healthcare system and people around them [9,10,11,48]. These connections combat feelings of isolation and loneliness [11,14,15,51]. Social media support encourages women to advocate for a diagnosis, access treatment, and obtain referrals to reduce out-of-pocket costs by facilitating empowerment, self-efficacy, and confidence [12,13,40]. Although connecting with other women is a key reason to access social media, endometriosis groups are on occasion considered unsafe spaces for key priority communities such as transgender or non-binary individuals, and women of colour [11]. Arguments and bullying on social media platforms can also cause distress for the individuals involved and those observing the posts [13]. It is important to note that social media groups may only be inclusive of women from a certain demographic such as developed countries, therefore missing out on important data from women in low socioeconomic circumstances who may not have access to social media.

Social media groups offer women a space to share their endometriosis experiences, resulting in both positive and negative emotions among authors and readers [11,13,15,16]. On a positive note, accessing others’ endometriosis experiences fosters empowerment and contributes to psychological wellbeing [14,16]. Furthermore, reading and sharing stories about symptoms and healthcare system interactions provides reassurance and validation [13,52]. However, accessing others’ negative experiences on these pages can also trigger psychological distress and discouragement in women, particularly when sensitive topics such as infertility and pregnancy are discussed [12,15].

Existing empirical research shows that women with endometriosis-like symptoms access social media to seek a better understanding of the condition [14,17,53]. Women express feeling as though educating themselves through social media and engaging in self-advocacy is the only way to receive sufficient treatment for endometriosis [51]. Women also access social media to seek information regarding coping mechanisms, symptomology, pathophysiology, epidemiology, diagnosis, management of symptoms, surgery, and treatment recommendations [11,16,40,47]. Social media content generates shared knowledge of lesser-known endometriosis symptoms that are often under recognised within the healthcare system, including migraines, nausea, muscle pain, inflammation, dizziness, and vision issues [12,41].

Women access social media to seek health information about endometriosis, including medications (pain and oral contraception) [9,13,16] and surgical interventions [9,41,54], with women often expressing distrust toward these treatments due to adverse side effects and insufficient information provided by healthcare professionals [41,47]. Due to women experiencing adverse side effects, they request alternative, non-evidence-based treatments on social media [41,53,55].

While current research is able provide an understanding of why women access social media in relation to endometriosis, no formal conceptual model has been applied to analyse this activity. Applying a conceptual model could help to organise these insights and deepen our understanding of women’s social media use. Implementing the IMB model allows the current study to explore how women seek to self-manage their endometriosis symptoms. By achieving an understanding of these behaviours, the current study aims to identify knowledge gaps that hinder women’s self-management of endometriosis.

### 1.4. Information–Motivation–Behavioural (IMB) Skills Model

The IMB model has been used in the present study to provide a structured, evidence-based framework for understanding health-related behaviours in endometriosis, particularly how women use social media to self-manage their conditions, which is an approach that has not yet been applied to endometriosis research. The IMB model conceptualises the factors influencing health behaviour adherence, including information, motivation, and behavioural skills [56,57,58]. Understanding these components provides important knowledge of how individuals self-manage their conditions [59]. This model has been used in the research of health conditions such as chronic obstructive pulmonary disease [60], osteoporosis [59], kidney transplant [61], stroke [62], atrial fibrillation [63], human immunodeficiency virus (HIV) [64,65,66], diabetes [67,68,69], and IBS [70], and is considered appropriate for the current study.

The information aspect of the model comprises an appropriate understanding of the condition and how it can be managed [56]. Motivation is divided into personal motivation, which includes attitudes and self-evaluation of behaviour, and social motivation, which relates to societal acceptance and the desire to conform to social norms [56]. Behavioural skills refer to strategies used by individuals to implement health behaviours effectively; these methods are influenced by available information and motivation [56]. Therefore, the IMB model is being used to analyse how social media shapes knowledge, motivates individuals, and provides management strategies related to self-management of endometriosis.

### 1.5. Aim and Research Questions

The primary aim of the current research is to explore how women with endometriosis-like symptoms use Reddit to self-manage their condition using the IMB model. The following research questions (RQs) were implemented to investigate this, with each question incorporating an aspect of the IMB model:

1. How are women with endometriosis-like symptoms utilising Reddit to seek information regarding their condition, as perceived through Reddit interactions? (RQ1).
2. How do women appear to be motivated to share on Reddit, regarding the personal and social impacts of endometriosis on their lives?
3. How do women with endometriosis-like symptoms describe and share behavioural skills on Reddit, including management strategies and resources? (RQ3).

## 2. Methodology

### 2.1. Procedure

#### 2.1.1. Ethical Considerations

Ethics approval for this study was granted by the Southern Queensland Ethics Committee (ETH2024-0277, 27 May 2024). Ethical considerations have been guided by previous studies utilising Reddit data [10,71,72]. Participant consent was not directly obtained per se; however, given Reddit’s public and anonymous nature, individuals consented to the research through means of posting on Reddit, as per the Privacy Policy [73], in line with studies such as Sanders et al. (2023) [74]. This study adhered to Reddit guidelines by replacing usernames with pseudonyms, paraphrasing excerpts, and excluding identifiable demographic information.

#### 2.1.2. Data Collection

It was determined that Reddit data were appropriate to include in this study, as this platform is public and allows users to remain anonymous through self-selected usernames. Reddit is considered the best-suited social media platform for data collection, as its anonymity fosters a supportive space for sensitive health discussions and mutual exchanges among individuals with shared experiences, thereby generating rich, raw, unfiltered data for the study to utilise, which is not available in other social media platforms. The subreddit r/endo is a popular platform for discussing endometriosis-related content, with approximately 66,000 members and 30 daily posts during the data collection period in June 2024. The r/endo subreddit was reviewed by two researchers (Author One and Author Two) to assess whether these posts were appropriate for analysis. It is important to acknowledge that Reddit data may exclude certain demographics, such as individuals from developing countries and older populations. This is because most users access Reddit from countries including the United States of America (USA), the United Kingdom, Canada, Germany, India, and Australia [75,76], and 75% of individuals using Reddit are between the ages of 18 to 49 [76].

As informed by prior Reddit research, a total of 200 of the most recent threads from the r/endo subreddit were aimed to be collected for the study [74,77,78]. Braun and Clarke (2013) [79] recommend the implementation of 200 data points for a qualitative study of this size, utilising pre-existing and text-based data, as this achieves generalisability and data saturation. To ensure that the data reflected current experiences and were representative of active Reddit users, the most recent threads were collected from r/endo, capturing the most recent discussions. To determine the generalisability of the r/endo data, the r/endometriosis and r/endo subreddits were reviewed by two researchers, as these were identified as the two most commonly used subreddits for endometriosis-related communication. The objective was to assess the appropriateness for analysis by observing the frequency of daily threads and their representativeness of the broader population. Upon observation of approximately 30 threads, the r/endo subreddit was considered representative of the population and appropriate to implement within the current study. Each thread within r/endo was collected for the initial dataset, including those immediately meeting an exclusion criterion. During data collection, it became apparent that numerous threads did not meet inclusion, and so to ensure that an appropriate amount of data were provided within the dataset, a further 30 threads were collected at the time of data collection.

Inclusion criteria were developed based on existing Reddit research such as Britt et al. (2023) [80], Garg et al. (2020) [58], and Jacques et al. (2023) [78]. Threads excluded from the dataset met the following criteria: (i) advertisements; (ii) research; (iii) if an individual specified they were under 18 years old; (iv) non-English posts; (v) not safe for work (NSFW) images; (vi) users who posted on behalf of another person with diagnosed or suspected endometriosis; and (vii) threads not consistent with the research aim and research questions. The inclusion/exclusion criteria are demonstrated in Table 1.

A total of 230 threads shared over a nine-day period (20–28 June 2024) were extracted from the r/endo subreddit on 28 June 2024. These data were collected manually, rather than using a Reddit extractor tool, as the researchers aimed to engage deeply with the data. These were then placed onto an Excel spreadsheet with the following headings: (i) username, (ii) date posted, (iii) date extracted, (iv) thread title, (v) thread, (vi) excluded, and (vii) included. It is noted that Reddit usernames were included within the initial dataset to observe instances where users posted multiple threads, and these were changed to pseudonyms after analysis. Additionally, only original posts, not comments, were included within the study to ensure the data remains a manageable size and to reduce the likelihood of data saturation occurring [74].

**Figure 1:**
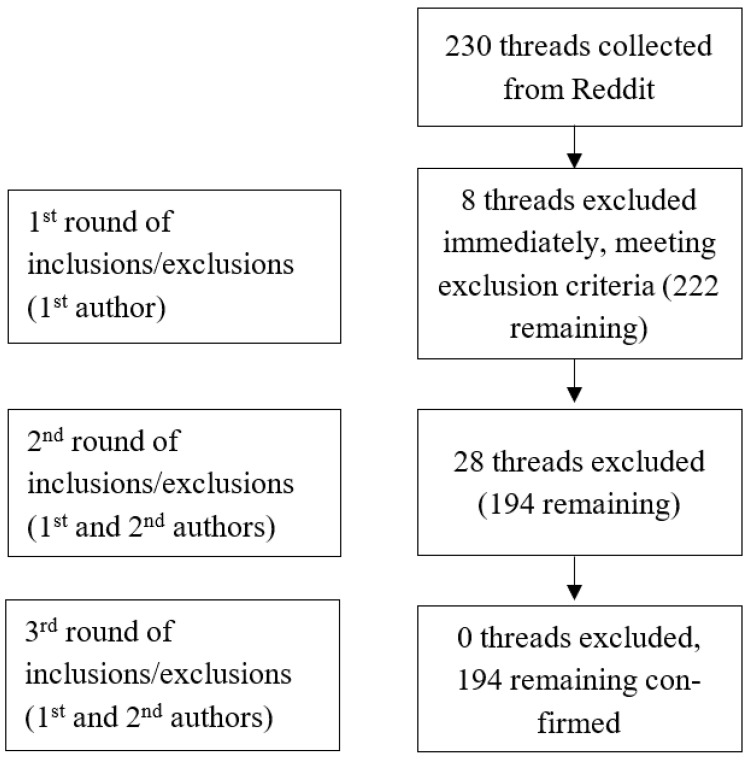
Reddit data exclusion process.

As detailed previously, initial exclusions were made immediately post-data collection if criteria were met, with eight threads removed by Author One. The secondary process involved examining each thread individually to determine if these were in accordance with the pre-determined themes, resulting in an additional 28 threads being excluded. Author Two then analysed the dataset to validate these exclusions. The final dataset comprised a total of 194 posts that were each assigned to at least one theme, with some threads being placed into multiple themes. It is noted that threads shared by those with a diagnosis and users with suspected endometriosis were included within the study, to ensure an enriched understanding of available data. This is depicted in Figure 1.

### 2.2. Data Analysis

A reflexive thematic analysis (TA) was carried out in accordance with Braun and Clarke (2013, 2021) [79,81], with modifications based on previous Reddit and social media studies [74,82] and IMB model research [61,83]. Reflexive thematic analysis is a six-step process that involves coding qualitative data for the purpose of identifying present themes [81]. This six-step process involves (1) familiarisation of the data; (2) creating codes; (3) arranging codes into themes; (4) generating and revising themes; (5) refining, defining, and naming themes; and (6) completing a written narrative [81]. Using TA allowed an understanding of central topics discussed on r/endo, thus gaining knowledge of how women on Reddit sought to self-manage their condition.

A deductive–inductive approach was applied to the TA, as guided by prior Reddit/social media [74,82], endometriosis [13,15], and IMB model research [61,83]. Themes were deductively pre-determined by the theoretical IMB mode—namely, information, motivation, and behavioural skills—and data was collected based on these themes. Codes and subthemes were identified based on the r/endo data (inductive). Themes were organised to each aspect of the IMB model. For the information aspect, the themes captured key types of endometriosis-related information sought by users. Motivation included personal and social circumstances appearing to motivate women to share their experiences on Reddit, and behavioural skills encompassed the management strategies/resources being shared.

Upon commencement of the analysis, the Excel dataset was placed into NVivo (version 14.23.3), a software package used for structuring and analysing qualitative data [84]. NVivo was utilised based on its use in prior research with comparable methodologies, making it well-suited for the current research [58,77,78,82]. NVivo was considered a vital element to include within the TA as it enabled the seamless organisation of data that may not have been provided by completing the analysis manually. To ensure inter-rater reliability, one researcher conducted the initial coding and thematic analysis, and a second researcher reviewed the codes and themes to ensure accurate analysis. Discrepancies were discussed until consensus was reached.

### 2.3. Researcher Positionality

It was imperative to ensure that the researchers were aware of how their experiences, presumptions, and expectations could influence the research process or outcomes. To ensure this, the authors—particularly myself (Author One), as I was the primary analyst of the data—engaged in ongoing reflexivity throughout the process, critically examining how our backgrounds and experiences could have an impact. I (Author One) identify as a white, university-educated female, coming from a middle-class background, with no experiences of a chronic health condition. I acknowledge that my privilege and lack of experience with chronic illness within the healthcare system may contribute to a limited understanding of what these women have experienced. Therefore, I consistently reflected upon and considered this factor to ensure that these women’s stories were told accurately, and with care.

Furthermore, the additional authors’ personal experiences were also considered, and included not having any lived experience with endometriosis and being from educated backgrounds. The research team comprised individuals with diverse cultural backgrounds and professional experiences. The authors dedicate their professional work to researching and supporting diverse individuals in both health and psychological research spaces. These perspectives contributed to a balanced and ethically grounded approach to the research.

## 3. Findings

**Figure 2:**
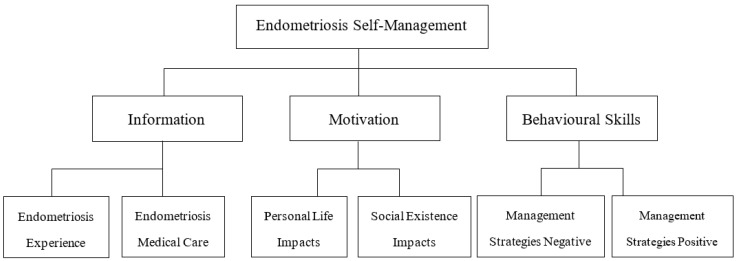
Thematic analysis map exhibiting three themes and six subthemes.

This study employed TA, guided by the IMB model, to explore how women seek to self-manage endometriosis on Reddit. The research started by identifying posts that appeared to be related to each facet of the IMB model, and then inductive TA was conducted within each of these datasets. Two subthemes were identified for each aspect of the IMB model: (1.1) *endometriosis medical care*; (1.2) *endometriosis experience*; (2.1) *personal life impacts*; (2.2) *social existence impacts*; (3.1) *management strategies negative*; and (3.2) *management strategies positive*. Identified themes and subthemes are provided within the thematic map in Figure 2.

Theme 1: Information

Findings from the current research showed that seeking information was a primary reason for users to access r/endo. Users requested information/advice on symptoms, fertility, and personal experiences, and this led to the creation of the endometriosis experience subtheme. Users additionally requested healthcare specifics such as medication, procedures, and self-advocacy within the healthcare system; this information is specific to endometriosis medical care.

Subtheme 1.1: Endometriosis Experience

Users sought information about endometriosis symptoms, with many undiagnosed individuals accessing r/endo to determine whether their symptoms aligned with the condition. One user mentioned the following:

*I’ve dealt with serious pain for years which I initially thought was something else… I have many endometriosis symptoms… I’m aware that something is not right with me, but I’m unsure if my symptoms are severe enough for endometriosis… Does this sound like endometriosis?*

Users enquired whether pain in the uterus, pelvis, hips, joints, back, breasts, and during intercourse was common. Pain was often linked with menstruation and/or chronic, debilitating some users for up to 10 years. One user asked the following question:

*… I’m living in daily pain… For years, I’ve had joint pain every day, especially during menstruation… I feel stiff, tired, and brain fog… Has anyone had the same experience?…*

Users additionally requested information on menstrual symptoms. Specific symptoms that were questioned included painful menstrual cycles, heavy blood flow (often for long periods of time), vomiting, fever, debilitating cramps (for the full menstruation cycle), bleeding from the rectum, and irregular periods. One user sought clarification by asking the following:

*During my cycle, I experience a pain as if my organs are about to burst… Is this endometriosis?*

Findings showed that users sought knowledge of lesser-known symptoms such as cysts, appendicitis, prolonged sickness, period flu, sciatica, exhaustion/dizziness, brain fog, hair loss, random spotting, odour, arthritis, and leg swelling. Users detailed continuously experiencing these symptoms for numerous years despite undergoing various diagnostic procedures without receiving a diagnosis (ultrasounds, MRIs, CTs, and laparoscopies) and enduring operative procedures to remove endometriosis tissue (laparoscopies).

Questions also included how endometriosis can impact a user’s fertility. Users asked questions about IVF success, treatments to increase fertility, and whether cysts affected conception. Many sought advice on how to discontinue endometriosis medication (e.g., contraception) to conceive whilst managing symptoms.

It was found that users sought an understanding of whether their physical and mental endometriosis experiences were shared, including navigating dating/long-term relationships and dyspareunia, helping loved ones understand endometriosis, and reducing resentment toward others who have not experienced the condition. Lastly, users requested advice on interactions within the healthcare system, specifically with GPs and emergency departments. One user recounted the following:

*I underwent a laparoscopy years ago with no endometriosis found… After self-advocating, I was diagnosed and put on medication, which caused heavy bleeding… Has anyone experienced this?*

Subtheme 1.2: Endometriosis Medical Care

Users queried birth control methods such as intrauterine devices (IUDs), contraceptive implants, and oral contraception brands such as Nikki, Movisse, Dienogest, Altavera, Apri, Yasmin, Seasonique, and Slynd. Questions included whether contraception had adverse side effects, how these impacted others, and how to transition to other medications (due to pregnancy) while simultaneously avoiding symptom flare-ups. Additionally, users sought information on whether hormone suppression would lead to further health implications whilst expressing feelings of concern. A user queried,

*… I was prescribed oral birth control to see whether this helped with symptoms… Has anyone used this, and what was your experience?…*

Users actively questioned the efficacy of prescribed medications for endometriosis. Users reported being prescribed medication by their GP or specialist and requesting further knowledge of the medication prior to implementation, due to their GP not providing this information. Prescribed treatments such as IBS and migraine medication, Hormone Replacement Therapy, and brands such as Leuprorelin, Myfembree, and Orilissa were questioned. After encountering adverse side effects from prescription medications, users enquired whether these were typical for the medication. One user asked the following question:

*… I’ve received numerous forms of treatment of Hormone Therapy… I have had the following changes: Hives and rashes… periods are the same… I am wondering what other people’s experiences are and would like to understand more about why these changes have occurred.*

Due to the side effects from contraception and medication, users sought information on alternative management strategies like pain patches, yoni steams, natural hormone balancing, and Cannabidiol (CBD) lotion, expressing concerns about long-term health risks from prescribed medications.

There were frequent enquiries about laparoscopies, MRIs, and CT scans, including their accuracy in diagnosing endometriosis, their impact on fertility, recovery time, and when intercourse can resume after undergoing such procedures. One user shared the following:

*I have a laparoscopy surgery booked in for a few weeks away and feel anxious… What was the laparoscopy like for you?…*

A few users sought advice on how to self-advocate within the healthcare system. Users requested advice on how to begin a dialogue with their GP about their concerns remaining unaddressed and symptoms being attributed to other conditions. Furthermore, users sought guidance on initiating conversation with their healthcare practitioners about the ineffectiveness of procedures such as laparoscopy, as well as requesting previously denied procedures such as MRIs and CT scans. As detailed by one user,

*… I have experienced endometriosis-like symptoms for years, with no endometriosis being found… I feel like my GP thinks that I am exaggerating or overreacting. How were you able to get your GP to take you seriously about endometriosis?*

Theme 2: Motivation

Users detailed how endometriosis impacted their personal and social existence, with this appearing to be a motivating factor to share their experiences on Reddit. Specifically, users detailed their personal experiences with endometriosis and the numerous ways it changed their overall lifestyle, mental health, and intimate relationships (*personal life impacts*), and social existence (*social existence impacts*).

Subtheme 2.1: Personal Life Impacts

Users reported how endometriosis removal procedures (laparoscopy) impacted their physical health, including causing severe pain, burning when urinating, nausea, dizziness, and gastrointestinal issues. Users described feeling unlike themselves following operative laparoscopy, MRI, and ultrasound procedures. Post-operative laparoscopy symptoms hindered daily activities, walking, and returning to work or education. Additionally, users reported their GP did not provide sufficient information about the procedural results and instructions on how to proceed. Numerous users underwent multiple laparoscopies (up to four) without pain relief. One user shared the following:

*… A few months after surgery, the pain I previously experienced returned… I feel so defeated. I am feeling frustrated as no one is taking my pain seriously…I am not looking forward to telling my GP… only to face defensiveness and gaslighting…*

Users detailed negative experiences with their GPs, with symptoms often dismissed, invalidated, or attributed to other factors, such as weight. This led to numerous visits to specialists and GPs, increasing the financial and emotional strain. One user described feeling “*severely depressed*” while waiting nine months for a specialist appointment and experiencing suicidal thoughts due to a lack of support during the process.

Debilitating symptoms and negative experiences within the healthcare system caused a myriad of negative feelings such as loneliness, worthlessness, hopelessness, misery, and burnout. Users described how endometriosis and its associated experiences, such as invalidation from their healthcare professionals, contributed to their stress, panic attacks, anxiety, depression, and post-traumatic stress disorder (PTSD). One user commenced anti-depressants because of the impact that symptoms and lack of support from healthcare professionals had upon their mental health. Numerous users expressed feeling as though the only way to cease their chronic pain was to end their life.

*… I have been in constant pain for years… My GP started looking into it in last year, and I’ve undergone tests… Everything came back clear, but the pain persists… My mental health has reached a breaking point… due to feeling like no health professional is helping me…*

In contrast, users detailed the positive contributions their GP had upon their diagnosis and treatment by advocating and educating themselves on the condition. This positively contributed to users’ physical and mental wellbeing.

Users shared how endometriosis significantly affected their ability to work, study, and care for themselves. Personal relationships were strained due to dyspareunia and irritability, with some avoiding relationships due to this. Users feared their partner might leave due to the increased levels of understanding and care they require. This resulted in feelings of loneliness, guilt, isolation, hopelessness, and frustration. One user shared the following:

*I’ve been taking my frustrations out on the important people around me.*

Subtheme 2.2: Social Existence Impacts

Users described how endometriosis affected their social existence. Many users frequently cancelled social engagements due to debilitating symptoms. These cancellations strained relationships. This led to feelings of guilt, with users struggling to explain their physical limitations and the effort required to attend planned outings. One user recounted the following:

*… I feel completely exhausted and in pain. I have promised to take my family member to the shops, but I’m unable to do that… I feel terrible and worry that I am a disappointment.*

Several users expressed feeling unable to relate to those without endometriosis. One user shared that their loved ones doubted the severity of their endometriosis symptoms. These circumstances resulted in users feeling lost, alone, and isolated, even if loved ones actively showed support. A user detailed the following:

*… I have a laparoscopy scheduled and my loved ones are convinced that no endometriosis will be found… If there is no endometriosis found, I will feel embarrassed and foolish.*

Endometriosis also impacted users’ ability to engage in social activities such as travelling, recreational sports, exercise, and attending the gym. Many users could no longer participate in these activities, which further contributed to their social isolation.

Theme 3: Behavioural Skills

Examination of the data revealed that users actively described the treatments used to manage endometriosis symptoms, with these being classified as behavioural skills. Users shared the negative and positive impacts that treatments such as contraception, medication, alternative, and post-procedural self-care had upon them. Two subthemes emerged, management strategies negative and management strategies positive, which referred to how different endometriosis management strategies negatively and positively impacted users’ experience with endometriosis.

Subtheme 3.1: Management Strategies Negative

Users shared their experiences with management strategies such as contraception, which caused negative impacts on their physical health, including consistent (severe) migraines, cramps, weight loss, acne, loss of appetite, fatigue, feeling faint, fever, diarrhoea, loss of menstrual cycle, rashes, prolonged bleeding during menstruation, worsened pain, IBS, body aches, bloating, mood swings, and depression. One user expressed feeling relieved once oral contraception treatment ceased, as it had significant impacts on their existence. An additional user detailed that their GP insisted on using oral contraception for treatment, as no other treatments were available. One user articulated their experience with the following statement:

*I was prescribed oral birth control… Over the past few months, I have been bleeding consistently, daily. I also experienced severe pain and heavy bleeding and was haemorrhaging.*

Additionally, users detailed using IUD contraception as a way to manage endometriosis; this led to adverse impacts, including insertion being debilitatingly painful, daily bleeding, light-headedness, extreme tiredness, feeling weak, breast pain and swelling, mood swings, body aches, back pain, depression, and feeling unlike themselves. These side effects were felt immediately post-insertion, and for numerous months, consistently. One user recounted the following:

*I had an IUD inserted… I recently began bleeding for almost a week. I am feeling pain and exhaustion. I felt my heart rate increase and am feeling nauseous…*

The data showed that users’ experiences with endometriosis medications included a lack of symptomatic relief. Specifically, pain medication such as Paracetamol and Ibuprofen did not effectively reduce daily and post-procedural pain, with some users taking the maximum daily dose without relief. Prescribed pain medications including Codeine and Oxycodone reduced everyday functioning, increased irritability, and caused dependency and eventual tolerance. Additionally, hormone injections and prescribed medications such as Omeprazole, Myfembree, and Primolut caused adverse side effects such as hives, stomach pain, and consistent menstrual bleeding. Furthermore, endometriosis-specific medications such as Orilissa triggered depression and anxiety. A user reported the following:

*… I have recently been in a lot of pain… Previously, taking Paracetamol and Ibuprofen would help to provide some relief, but now I need to take the maximum amount numerous times a day, with the pain persisting…*

Subtheme 3.2: Management Strategies Positive

Findings from the study showed that users described the positive contributions that oral contraception brands such as Yaz, Altavera, and Seasonique brought to the management of their condition. Oral contraception reportedly reduced menstrual-related pain by allowing users to skip their menstrual cycle. Users additionally detailed feeling anxious about potentially ceasing their use of oral contraception in the future to conceive, as it considerably alleviated their pain. Often, endometriosis symptoms would promptly re-emerge once oral contraception was ceased, causing debilitating pain. One user mentioned becoming clinically depressed and experiencing suicidal ideation once oral contraception was discontinued. A user detailed the following:

*… For the past few years, I have been managing my symptoms through contraception, which has made my life way more manageable… However, I plan to start trying to conceive in the next two years… Out of curiosity, I decided to try the placebo week of my contraception, and my pain intensified…*

A small percentage of users shared strategies for managing daily endometriosis pain and post-procedural symptoms, including the use of pain patches, Ibuprofen, Paracetamol, and medically prescribed brands such as Myfembree, Orilissa, Oxycodone, and Tramadol. Endometriosis symptoms often re-occurred after medically prescribed treatments were ceased. As shared by one user,

*… My endometriosis symptoms had decreased for the past few years whilst I was on Myfembree… Since I have tried a new medication for the past few weeks, my endometriosis pain has come back, with other severe symptoms such as cramps and headaches…*

Users additionally discussed alternative strategies, after medication proved to ineffectively manage their symptoms. Alternative treatments included an anti-inflammatory diet, cortisol cream, CBD, vitamin supplements (Chinese herbs, Omega-3, Vitamins B and D, castor oil, and anti-inflammatory vitamins), electrolytes, and working on physical health through Pilates and weightlifting. Users reported improvements in mental health after regularly exercising and participating in talk therapy. An additional remedial method included physical therapy, with users sharing both positive and negative experiences associated with this. One user detailed the following:

*… Prescription medications do not work for me… I tried cannabidiol (CBD) during my last period, and after using this, I did not experience pelvic/back pain and cramping…*

Users additionally shared post-procedural self-care strategies. This included the use of exercise training equipment to enable easier, strain-free movement, as well as a pregnancy pillow for comfort.

## 4. Discussion

The current study contributes to the existing literature through implementing the IMB model to explore how women with endometriosis-like symptoms use Reddit to self-manage their conditions. To our knowledge, the IMB model has not been utilised in prior endometriosis research to examine this condition.

A key finding arising from the present study included that the key factor behind women posting on Reddit was to seek endometriosis-specific information and/or advice. Findings extend previous literature such as Goel et al. (2023) [41] and van den Haspel et al. (2022) [15], demonstrating that women primarily access social media to obtain essential information on the endometriosis condition. Specifically, women sought understanding of common and lesser-known symptoms such as dizziness, inflammation, menstrual flu, appendicitis, gastrointestinal issues, nerve flares, hair loss, and bone/joint pain, which is consistent with the findings of Fruchart et al. (2023) [22] and Sbaffi and King (2020) [12]. Women reported that their uncommon endometriosis symptoms were unrecognised and invalidated by healthcare practitioners, despite being preliminary indicators of the condition [41]. In accordance with Shoebotham and Coulson (2016) [13], women additionally accessed social media to understand whether their endometriosis experiences were common. Women expressed seeking this endometriosis-specific information on social media due to insufficient accessibility of information and support being available within the healthcare system [9,40,41,53,85].

Notably, women actively sought information on pain and endometriosis medications [10,16,41]. This included questioning whether certain medications were recommended by others, common side effects, and other users’ experiences with these. Women detailed accessing social media due to their treating doctors being inaccessible for clarification, prescribed treatments being ineffective/ceasing efficacy, and adverse side effects occurring after use.

Additionally, information on laparoscopy and hysterectomy surgeries was requested [9]. Questions consisted of whether symptoms alleviated after certain procedures, recommendations based on others’ experiences, and post-surgery aftercare [13,47,53]. Some women reported turning to social media for information due to insufficient guidance or support from their GP. Given that contraception, pain medication, and laparoscopy are primary methods of treating endometriosis, this knowledge deficit is considered detrimental to the population [34]. These queries contain information that should be addressed by healthcare professionals. By implementing social media recommendations, women may be engaging with inaccurate knowledge and symptom management, as research such as Arena et al. (2022) [86], Isaac et al. (2024) [45], and van den Haspel et al. (2022) [15] highlight the presence of misinformation.

Consistent with previous research, individuals actively pursued guidance on self-advocacy within the healthcare system. Particularly, women accessed social media as a final resort due to experiencing a lack of validation and empathy, and the normalisation of pain within their respective healthcare systems [4,10,31,41,42]. These findings demonstrate that women turn to social media due to feeling disempowered within their respective healthcare systems when attempting to access care and treatments. Women expressed that the support from the online community benefitted their mental health, helping to alleviate stress and loneliness while fostering a sense of empowerment. In line with these findings, Arena et al. (2023) [87] found that women who were evaluated by gynaecologists for possible endometriosis were highly anxious and “probably have searched the internet extensively for their long-answered doubts” (p. 5). The current study’s findings contribute to a common theme across endometriosis literature, specifically that women with endometriosis-like symptoms feel invalidated and unsupported by their respective healthcare systems, despite consistently advocating for appropriate care [9,22,40,53,85]. Women who turn to social media due to this lack of support may be vulnerable to misinformation that could influence their treatment decisions [86]. Additionally, these platforms may contribute to a harmful cycle by exacerbating anxiety and depression associated with chronic pain, particularly when users are exposed to others’ negative experiences, as noted in previous research [12,13,15].

An unexpected finding within this study included women’s post-laparoscopy experiences. Experiences following operative laparoscopic procedures (removal of endometriosis lesions) included only short-term relief from pain, and increased pain compared to before [21,25,34,88], which is inconsistent with the findings of Bastu et al. (2020) [89] and Rindos et al. (2020) [90]. Additionally, women required numerous laparoscopic operative procedures carried out within a short time frame due to symptom reoccurrence, with this contributing to financial stress and impacting mental wellbeing by increasing feelings of distress and anxiety [13,28,34,35,54,91]. An additional finding not reported in previous research was that women experienced feelings of loneliness and disconnection from their former selves following an operative laparoscopy, highlighting an aspect of mental health that is effected yet under-researched.

Extending on previous research such as Goel et al. (2023) [41] and Lindgren and Richardson (2023) [10], women accessed social media to detail negative interactions and experiences with healthcare professionals. These encounters included a perceived disregard of symptoms, gaslighting, and normalisation of pain [4,9,11,12]. In line with previous literature, individuals with suspected endometriosis stated that their symptoms were attributed to other factors such as weight and healthcare conditions including IBS [31,39,43,51]. Due to this, women felt as though they had to self-advocate to receive a diagnosis and treatment [38]. This self-advocation was associated with feelings of frustration and exhaustion, impacting their everyday existence, with social media pages providing relief from burnout, in line with Holowka (2022) [11]. The lack of support and treatment options was seen as problematic throughout the dataset, with two posts even reflecting on suicidal ideation as a result of this lack of support [9,41]. This perceived lack of support within the healthcare system is considered detrimental, as it leads women to believe their debilitating symptoms are unmanageable, generating feelings of hopelessness.

Contrastingly, posters additionally shared the positive impact that healthcare practitioners had upon their existence. Practitioners who educated themselves on the endometriosis condition and advocated for their patients positively contributed to timely diagnosis, effective treatments, and overall wellbeing [12,37,39]. These findings highlight the significant role health practitioners play in providing women with knowledge, education, and support.

Consistent with previous literature such as Missmer et al. (2022) [28], Wilson et al. (2020) [16], and Wu et al. (2023) [47], women actively shared their experiences with prescribed treatments on social media. Specifically, women who communicated their experiences with contraception (both oral and IUD) largely detailed adverse side effects that impaired everyday functioning. These included migraines, pain, consistent bleeding for months, appetite change, no longer feeling like themselves, depression, cramping, tiredness, and inflammation [88]. Findings align with Wu et al. (2023) [47], which specified that 80% of social media posts addressing contraception reported adverse side effects. Despite these findings supporting Wu et al. (2023) [47], limited research is available that explores women’s subjective experiences of using contraception to manage endometriosis. Due to these adverse side effects, women within the current study attempted to access alternate endometriosis treatments from healthcare professionals, but were informed that contraception was the only treatment available for the condition. The current study expands on the previous literature by highlighting that women with endometriosis consider contraception to be an inefficacious treatment for endometriosis, despite this being one of the only treatments available [47].

Furthermore, findings highlighted women’s negative experiences with pain and endometriosis-specific medications [16,28]. Specifically, heavy pain medications were often avoided as they led to decreased alertness, thereby reducing users’ capacity to perform daily routines such as attending work and completing household tasks, as validated by Grogan et al. (2018) [24] and Peterson et al. (2023) [5]. Although endometriosis-specific medications such as Myfembree and Orilissa provided relief for some, these had long-term physical implications, including decreasing bone density, which required treatment to cease [34,40]. Women expressed feeling as though they had to choose between adverse short- and long-term medication-induced side effects or debilitating endometriosis symptoms [24,31]. Consistent with Ellis et al. (2022) [34] and Wu et al. (2023) [47], women communicated distrust of and resistance to prescribed medical treatments because of these side effects. Furthermore, women articulated feeling distressed as endometriosis treatments could not be administered when planning for pregnancy, thus forcing women to feel as though they are need to choose between having a child or experiencing endometriosis symptoms [34]. The findings indicate that the currently available endometriosis treatments (contraception, pain, and endometriosis-specific medication) have adverse short- and long-term side effects on women.

Consistent with Adler et al. (2024) [40] and Goel et al. (2023) [41], women often implemented alternative endometriosis treatments for symptom management due to experiencing adverse side effects from medical treatments. These included non-empirical alternatives such as vitamin supplements, anti-inflammatory diets, and electrolytes. An unexpected finding from the current study included the use of alternate management strategies such as CBD oil and Chinese herbs, and their ability to successfully reduce users’ acute and chronic pain; additionally, women expressed improvements in their mental health after implementing exercise and talk therapy. These findings support Wilson et al. (2020) [16], emphasising the efficacy of alternate methods in reducing endometriosis-related pain and symptoms.

### 4.1. Implications

This study offers valuable insights into women’s experiences with endometriosis, highlighting how the healthcare system can tailor support to this population. To improve accessibility of care, the healthcare system must address gaps in endometriosis medical care by making necessary adjustments to existing processes [11,12]. Key improvements include providing comprehensive knowledge and education about the condition [15,41], offering validation for women’s experiences [10,11,12], and enhancing women’s access to efficacious treatment options by ensuring that such treatments are appropriately researched and prescribed [12]. Additionally, the current study underscores the importance of providing holistic multidisciplinary support systems, including counselling and psychological support for women with endometriosis, so as to mitigate the psychological distress that is caused by the condition and the lack of support within the healthcare system. Based on our findings, to address the current gaps in the healthcare system, it is recommended that women experiencing endometriosis-like symptoms should have improved access to timely diagnoses and a broader range of treatment options. Ensuring that care is more accessible and aligned with patients’ preferences will enhance the effectiveness and satisfaction of treatment.

Through contextualising how women self-manage their condition, the current study was able to develop a preliminary IMB model for endometriosis. The informational component includes essential knowledge of symptomology, common experiences, and medical treatments. The motivational factor comprises increased support within the healthcare system, in addition to accessing others with similar experiences. Furthermore, the behavioural skills facet includes efficacious medical treatments that support women’s autonomy and wellbeing. It is recommended that future research explore the utility of other theoretical frameworks (e.g., Leventhal’s Parallel Processing or Commonsense Model [92]) in optimising self-management of health conditions, as well as exploring other health communication approaches (e.g., narrative) [92,93] specific to other health contexts/target groups, to further build on these findings.

Furthermore, the current research study has identified the importance of utilising digital health platforms as support mechanisms for women with endometriosis. It highlights the need to develop and disseminate health information on digital platforms that is accessible, credible, and relatable for women [15,16]. As this study indicates a barrier of accessible care in the healthcare system, providing information and potential interventions on digital platforms may provide accessibility to women with endometriosis, whilst minimising the spread of online misinformation [13,15].

### 4.2. Limitations

Although the current study provides insight into how women self-manage their condition, a potential limitation is that the data were collected from a single source, Reddit. Therefore, the data could potentially be limited to a subset of the population, likely younger women (18–49 years old) who are more motivated to seek information on Reddit [76], or those reluctant to share their symptoms with a healthcare professional. Additionally, the data may not account for non-English-speaking cultures, age, culture, geographic location, and co-morbidities, which may modify how users manage and experience their condition. Accessing Reddit data could potentially lead to bias as users tend to access social media pages to report adverse or concerning experiences, with this factor needing to be considered when positioning the current research in relation to existing studies [88]. It is important to note that while Reddit data provides valuable insight into users’ experiences and support-seeking behaviours, this platform represents a communication pathway that may not reflect credible or accurate information regarding symptoms, treatments, or management of the condition. Although Reddit data may not enable the study to capture a complete picture of endometriosis, the researchers still consider this to be a meaningful sample.

### 4.3. Future Research

The current study suggests that future research explore how women with endometriosis can be supported in the self-management of their condition within the Australian healthcare system. This could be achieved by implementing the IMB model in a direct context such as interviewing Australian women with endometriosis. Refining the current endometriosis IMB model by conducting further research will provide insights into the obstacles preventing women from effectively self-managing their condition. Once this has been established, further research can create mixed methods-based pilot study with health resources to observe whether, and how, the self-management of endometriosis can be increased.

The present study has highlighted the importance of utilising social media to allow women’s voices to be heard. Furthermore, it is recommended that future research triangulate Reddit findings with interviews, surveys, and analyses of other social media platforms to increase validity and determine whether patterns are generalisable to other women with endometriosis, ideally using a mixed methods design to provide more comprehensive analysis. Furthermore, it could be of further interest to include a focus on social media influencers and the role they play in women’s experiences.

## 5. Conclusions

In conclusion, the current study aimed to view how women with endometriosis-like symptoms self-manage their conditions on Reddit. Women’s subjective accounts on social media highlight a gap in endometriosis knowledge and accessible care within their respective healthcare systems. It additionally became clear that currently available endometriosis treatments have implications upon women’s physical and mental wellbeing. By exploring how women with endometriosis use social media, in accordance with the IMB model, the current study provides insights into how women can be supported in the self-management of their condition, particularly by the healthcare system. In line with these findings, it is suggested that insights from social media communities be integrated with traditional healthcare interventions, potentially providing women with enhanced support by enabling accurate education, validating symptoms, facilitating access to peer communities, and delivering efficacious medical treatments. Ultimately, integrating these insights can help ensure that the healthcare system provides more comprehensive support, so that women with endometriosis-like symptoms no longer feel dismissed, gaslit, or forced to advocate for themselves to receive appropriate care.

## Figures and Tables

**Table 1:** Inclusion and exclusion criteria for Reddit thread selection.

Inclusion	Exclusion
- Posts relating to experiences of endometriosis. - Posts relevant to the study aim and research questions.	- Advertisements. - Research recruitment of data collection requests. - Posts by individuals under 18 years of age. - Non-English posts. - NSFW image-based posts. - Posts written on behalf of another person. - Threads not consistent with the study aim or RQs.

## Data Availability

The data that support the findings of this study are available from the corresponding author upon reasonable request. Due to ethical restrictions, some data may not be publicly shared.
